# Polyvinyl Alcohol/Sodium Alginate Hydrogels Incorporated with Silver Nanoclusters via Green Tea Extract for Antibacterial Applications

**DOI:** 10.1080/15685551.2020.1804183

**Published:** 2020-08-05

**Authors:** Tianwen Wang, Fang Zhang, Rui Zhao, Can Wang, Kehui Hu, Yi Sun, Constantinus Politis, Amin Shavandi, Lei Nie

**Affiliations:** a College of Life Sciences, Xinyang Normal University, Xinyang, China; b College of Life Science & Technology, Huazhong University of Science and Technology, Wuhan, China; c College of Chemistry and Chemical Engineering, Xinyang Normal University, Xinyang China; d Department of Mechanical Engineering, Tsinghua University, Beijing, China; e Department of Imaging & Pathology, University of Leuven and Oral & Maxillofacial Surgery, University Hospitals Leuven, Leuven, Belgium; f BioMatter Unit - École Polytechnique De Bruxelles, Université Libre De Bruxelles, Brussels, Belgium

**Keywords:** Silver nanoparticles, antibacterial hydrogel, green synthesis, reductive metabolites, tea extract

## Abstract

Silver-based nanoparticles and biomaterials have extensive biomedical applications owing to their unique antimicrobial properties. Thus, green and facile synthesis of such materials is highly desirable. This study reports an antibacterial hydrogel based on polyvinyl alcohol/sodium alginate network with the incorporation of silver nanoparticles (AgNPs), which is greenly synthesized by reductive metabolites obtained from the leaves of green tea. The ‘flower-shape’ AgNPs were acquired, it formed a mono-disperse system with a distinct uniform interparticle separation. The average size of AgNPs varied from 129.5 to 243.6 nm, which could be regulated by using different volumes of the green tea extract. Zeta potentials of the AgNPs were from −39.3 mV to −20.3 mV, indicating the moderate stability of the particles in water. In the next stage, the antibacterial polyvinyl alcohol/sodium alginate hydrogels were fabricated by incorporating prepared AgNPs. Scanning Electron Microscopy (SEM) images showed that the porous structure was obtained, and Energy Dispersive X-Ray (EDX) analysis confirmed that the AgNPs were uniformly dispersed in the polymer network. The hydrogels exhibited superior water absorption properties, which were characterized by a high swelling ratio (500–900%) and fast equilibrium. The hydrogels also exhibited good antimicrobial activity in assays with Gram-positive bacteria *Escherichia coli* and Gram-negative bacteria *Staphylococcus aureus*. To sum up, a process for the green preparation of antibacterial hydrogels based on AgNPs derived from tea leaves as a conveniently available cheap local agricultural product was established.

## Graphic Abstract

**Figure:**
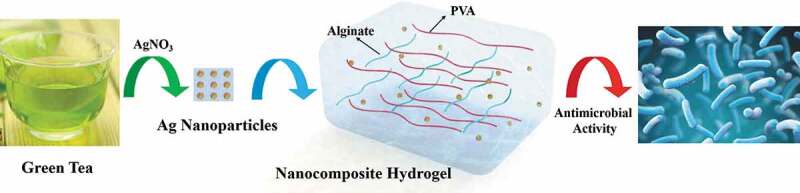

## 1 Introduction

Metal nanoparticles exhibit exciting properties such as electronic, magnetic, and optical properties with antibacterial activities, and are extensively applied in biomedical fields, including imaging and sensing, drug and gene delivery systems, and the isolation and detection of pathogens [1–3]. Silver nanoparticles (AgNPs) with high stability, high specific area, dynamic antimicrobial properties and ease of surface functionalization [4] have been extensively used as the antimicrobial agents in fighting against multidrug-resistant microorganisms [5,6], cleaning and disinfecting agents for medical devices (e.g., non-disposable filters), and as the carrier agent for drug delivery [7]. Generally, AgNPs could be synthesized by chemical reduction, photoreduction, laser synthesis, and other methods [8–12]. However, these methods are usually time-consuming and labor-intensive. Although reducing and protective agents are useful for synthesizing stable and non-aggregated AgNPs, some agents are biological hazards with environmental toxicity [13].

Recently the development of green synthetic AgNPs with excellent dispersibility and thermal stability has evolved into an active research area. Green solvents, ecologically benign reducing agents, and safe stabilizers or dispersants are three critical factors to consider in the green synthesis of AgNPs. It was reported that the phytochemicals in tea play a dual role as an active, reducing agent to reduce gold, silver, and palladium and also as stabilizers to provide a stable coating on the surface of nanoparticles in a one-pot process [14].

Tea is a popular and widely consumed beverage throughout the world, which is essentially the extract of leaves and buds of tea plants (*Camellia sinensis*) [15]. The tea metabolites are water-soluble with low toxicity, which exhibits great potential in drug development [16–18]. Several scientific studies have shown that tea contains high levels of antioxidant polyphenols, such as flavonoids and catechins [19–23]. These polyphenolic compounds can eliminate the dangerous free radicals such as superoxide anion radicals (O_2_∙), hydrogen peroxide (H_2_O_2_∙), and hydroxyl radicals (HO∙), thereby preventing the progression of various diseases such as cancers [24,25] (e.g., prostate gland cancer [26], lung cancer [27]), osteoporosis [28], cardiovascular diseases [29] and Parkinson’s disease [30], abdominal aortic aneurysm [31].

The bactericidal activity of AgNPs depends on their stability in the growth medium. Due to the large size and slow release rate of silver ions, the AgNPs synthesized by tea leaf extract had a lower antibacterial activity against *Escherichia coli* [4]. The combination of AgNPs with water-soluble biopolymers such as sodium alginate, Arabic gum, starch, gelatin, carboxymethyl cellulose, etc., were used in the production of multifunctional biocompatible polymeric silver nanocomposites or hydrogels [32–36]. Sodium alginate (SA) is a natural linear polysaccharide (β-D-mannuronic acid and α-L-guluronic acid linked by α-1, 4 bonds), which is extracted from the cell wall of brown algae. Such a unique structure confers its capacity to absorb large amounts of water to form hydrogels [37]. Also, Alginate contains the functional carboxylates group that can simply dissociate in the aqueous phase to carry negative charges. Different from alginate, polyvinyl alcohol (PVA) is a synthetic water-soluble polymer with non-ionic, polyhydroxylated, and biocompatible properties, which is often used as a stabilizer during the synthesis process of nanoparticles [38–42]. Also, PVA is non-toxic and highly biocompatible [43,44]. Although PVA constitutes an environmental concern because of its low biodegradability if PVA is directly disposed to the environment, the application of PVA in the biomedical area is still generally acceptable. In recent years, PVA-based hydrogels have been used as ideal polymeric wound dressing membranes for the treatment of skin wounds [45]. Such as Golafshan et al. fabricated laponite reinforced PVA-alginate nanohybrid hydrogels, which could promote hemostasis and was recognized as a desirable candidate for wound healing process [46]. It was shown that the biopolymer-protected AgNPs exhibited potential antibacterial activity [47]. Sun *et al*. used aniline, carboxymethylcellulose, and sodium alginate to synthesize CMC@AgNPs and SA@AgNPs [4]. Velusamy *et al*. used a microwave-assisted irradiation method to synthesize antibacterial hydrogels based on different concentrations (0.5, 1, 1.5, 2%) of carboxymethyl-cellulose, aniline, sodium alginate, sliver nanoparticles [47]. In another study, Narayanan *et al*. immobilized borate-stabilized silver nanoparticles as nanofillers in double crosslinked polymers composed of different proportions of PVA and SA. Narayanan *et al*. have already proved that the synthesized PVA/SA/AgNPs nanocomposites exhibited good antibacterial activity against *E. coli* O157:H7 with potential application as safe food packaging materials to extend the shelf life of food [48].

In this paper, we reported the use of tea extract as a reducing agent to synthesize natural molecules-stabilized AgNPs. Then the PVA/SA hydrogels containing the synthesized AgNPs were fabricated. The physicochemical properties of the AgNPs and the hydrogels were characterized, and the antimicrobial activities of PVA/SA/AgNPs hydrogels against Gram-positive bacteria *Escherichia coli* and Gram-negative bacteria *Staphylococcus aureus* were evaluated. Despite the fact that AgNPs can be independently used as an antimicrobial agent, and AgNPs are also the primary component for the antimicrobial hydrogel reported here, the antimicrobial hydrogels will extend the applications of AgNPs because of the unique properties of these composites. Moreover, the preparation process is environment-friendly. This study will also broaden the applications of tea leaves and an abundantly available local agricultural product.

## 2. Materials and Methods

### 2.1 Materials

Petroleum ether (C_5_H_12_), acetone (CH_3_COCH_3_, 95%), ethanol (C₂H₆O, 95%), polyvinyl alcohol ((C_2_H_4_O)_n_), 99%), hydrochloric acid (HCl, 36–38%) from Sinopharm Co., Ltd (Shanghai, China). Sodium chlorite (NaClO₂, 80%) and sodium alginate ((C_6_H_7_O_6_Na)_n_) were purchased from Sigma-Aldrich Chemical Reagent Co., Ltd. Potassium hydroxide (KOH, 90%) was from Ron Reagent Co., Ltd. Silver nitrate (AgNO_3_, 99.8%) was a product of Tiangen Reagent Co., Ltd., (Beijing, China) and calcium chloride (CaCl₂, 96%) of Macklin Reagent Co., Ltd (Shanghai, China). Green tea (Xinyang Maojian Tea, Xinyang, China) was provided by Henan Key Laboratory of Tea Plant Biology. Gram-negative *Escherichia coli* (ATCC 25,922) and Gram-positive *Staphylococcus aureus* (ATCC 6538) used for antimicrobial activity assay were lab stocks [49]. All other chemicals were purchased from China National Medicines Corporation Ltd., (analytical grade) and used without further purification.

### 2.3 ‘Green’ synthesis of silver nanoparticles (AgNPs)

The silver nanoparticles (AgNPs) were synthesized by following the method previously reported with minor modifications [50]. In preparation of the tea extract, 1 g of tea leaves were boiled in 50 mL of distilled water for 30 min. After filtration with four-layered cheesecloth, the filtrate was centrifuged at 5000 g for 10 min to obtain a clear solution. 1 mL of 0.1 M AgNO_3_ was mixed with 10 mL of the tea extract and 10 mL of distilled water. Subsequently, the mixture was stirred at 700 r/min to ensure thorough mixing and then kept still at room temperature (RT). The color of the mixture was changed from light brown to green, indicating the formation of AgNPs [8,51,52]. The process was repeated by using different volumes of the tea extract (10, 5, 3, and 1 mL), and the synthesized nanoparticles were correspondingly designated as T-AgNPs 1–4, as shown in **Table 1**.

**Table 1.** Silver nanoparticles prepared via chemical reduction by the green tea extract

Samples	Green tea extract^1^	AgNO_3_ (0.1 M)
T-AgNPs-1	10 mL	1 mL
T-AgNPs-2	5 mL	1 mL
T-AgNPs-3	3 mL	1 mL
T-AgNPs-4	1 mL	1 mL

1: Green tea extract: 20 g/L

### 2.4 Synthesis of silver-based nanocomposite hydrogel

Polyvinyl alcohol (PVA), sodium alginate (SA) are widely used to prepare biocompatible materials, because of their excellent biocompatibility, low-cost, non-toxicity, and convenient availability [53]. Therefore, silver nanocomposite hydrogels were fabricated by using PVA, SA, and the AgNPs in this study. Hydrogels fabricated with the AgNPs prepared with different recipes (**Table 1**) were accordingly designated as Hydrogels-1, Hydrogels-2, Hydrogels-3, and Hydrogels-4. First, the PVA solution was prepared by dissolving 0.2 g of PVA powder in 20 mL of hot distilled water (90 °C). Then 0.2 g of SA powder was added to the cooled PVA solution and stirred at 50 °C until the mixture became homogeneous. After then, the AgNPs in the water was added and stirred under the dark environment for 30 min. Regarding that CaCl_2_ is one of the most frequently used agents to ionically crosslink alginate, here CaCl_2_ was used as crosslinker. The obtained solution was poured into a 24-well plate, and after cooled to RT, a few drops of CaCl_2_ solution (5%, w/v) were added dropwise. After 24 h, the excess CaCl_2_ solution was drained out, and hydrogels were obtained. The hydrogel was washed three times with deionized water, and the surface water was subsequently removed. After overnight storage in a freezer −20 °C, the samples were freeze-dried to give rise to the formation of Ca^2+^ crosslinked PVA/SA/AgNPs nanocomposite hydrogels.

### 2.5 Transmission electron microscopy (TEM) characterization

The morphology of AgNPs prepared with tea extract was characterized using Transmission Electron Microscopy (TEM, Tecnai G2 F20) operating at 200 kV. For TEM sample preparation, 0.1 mL of AgNPs was ultrasonically dispersed in 5 mL of distilled water, and a drop of the particle suspension was dropped onto a carbon-coated copper mesh and dried at RT. The average size of the AgNPs was determined using ImageJ software using over 10 TEM images, at least.

### 2.6 Dynamic light scattering (DLS) and zeta potential characterization

The size distribution of the hydrated nanoparticle was determined by a Zetasizer (Malvern Instruments, Zetasizer NanoZS, 3000E, UK). Before the test, the sample was subjected to multiple ultrasonication and centrifugation. The test was conducted with triple-distilled water of pH = 7 as a reference. The zeta-potential (Malvern Instruments, Zetasizer 3000E, UK) was analyzed to probe the surface charge of AgNPs.

### 2.7 Fourier transform infrared (FT-IR) characterization

Fourier Transform Infrared (FT-IR, PerkinElmer, Spectrum 2) measurements were carried out to identify the functional groups that were bound distinctively on the AgNPs and silver-based nanocomposite hydrogels [54]. Samples for FT-IR measurement were prepared by mixing 1% (w/w) specimens with 100 mg of potassium bromide powder and pressing the mixture into a sheer slice. The resolution was 16 cm^−1^ in the wavenumber region of 400–4000 cm^−1^.

### 2.8 Scanning electron microscopy (SEM) characterization

The surface morphology of the silver-based nanocomposite hydrogels was observed by using Scanning Electron Microscopy (SEM, Hitachi, Japan, Model S-4800), the samples were cut into thin slices using a razor blade and placed on double-sided tape to coat the sample with platinum. Under backscatter (BSC) and secondary electron (SE) modes, the surface morphology of the samples was observed under SEM with different magnification. Besides, the Energy Dispersive X-Ray Spectroscopy (EDX) was operated during SEM testing to confirm the elements in hydrogels and the disperse situation of AgNPs in hydrogels.

### 2.9 Swelling behavior of the hydrogels

The equilibrium swelling ratio (ESR) of the hydrogel after drying was measured by a conventional gravimetric method. The weight of the dried hydrogel sample was accurately weighed, and then the dry samples were soaked in deionized water (Millipore water) at RT to reach a state of balanced swelling of each angle. Then, the sample was taken out from the water, and the free water on the surface of the hydrogels was wiped out with a tissue paper before the second weighing. ESR could be calculated based on the following equation:

Where *ms* was the mass (g) of the hydrogel in the equilibrated swollen state; *md* was the mass (g) of the dried hydrogel before swelling.

In the study of the swell kinetics of the hydrogel, the hydrogel soaked in water was weighed at intervals (*t*), and the swell rate at the sampling time was calculated.

### 2.10 Antimicrobial activity assay

In the antimicrobial activity assay of prepared hydrogels, two typical bacterial strains of Gram-negative *E. coli* (ATCC 25,922) and Gram-positive *S. aureus* (ATCC 6538) were used. Single overnight colonies of *E. coli* and *S. aureus* revived from the glycerol stocks on the Luria-Bertani (LB) agar plated were transferred to tubes with sterilized liquid LB to make a seed culture by growing at 37 °C with shaking (180 r/min) for 15 h. The actively growing seed culture was diluted into flasks containing fresh LB medium and cultured under the same condition. The growth was monitored by checking the optical density at 600 nm (OD_600_). When OD_600_ reached 0.6 (corresponding to the mid-exponential phase), the broth was diluted to 2 × 10^6^ CFU mL^−1^ with a sterile 0.9% NaCl solution. Cell suspension (50 μL) was evenly spread onto LB agar with sterile glass beads (90 mm). The hydrogels were prepared in a mold to a cylinder with the same diameters to ensure the hydrogels utilized in antimicrobial activity assay were comparable in weight and shape, and the hydrogels were then cut into slices with equal thickness using a sharp blade. These slices were carefully placed on the agar surfaces spread with the bacterial cells. Three replicates were used for each strain and hydrogel. Finally, after incubation at 37 °C for 24 h, the growth of bacteria on the plates was recorded and analyzed.

### 2.11 Statistical analysis

Each experiment was performed in triplicate if without a particular explanation, and all results were expressed as means ± SD. Statistical analyses were performed via the SPSS software package. Levene’s test was performed to determine the homogeneity of variance for all the data, and then Tamhane Post Hoc tests were performed for the comparison between different groups. Different *p* values of <0.05 (*), <0.01 (**), and <0.001 (***) were considered as statistically significant.

## 3. Results

### 3.1 Preparation of the AgNPs and AgNPs containing hydrogels

**Figure 1.**
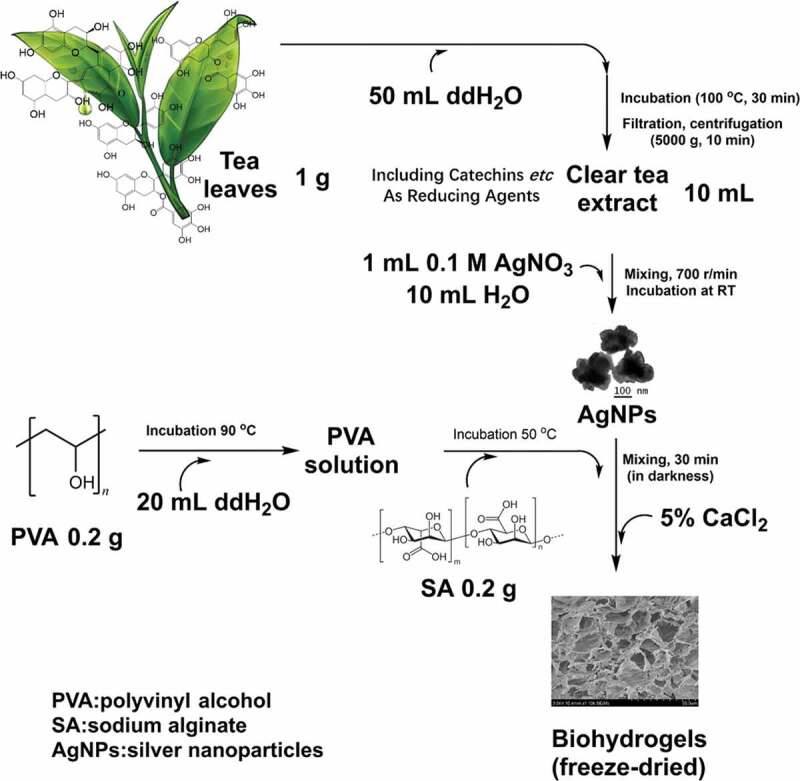
A green preparation process of the hydrogels. Due to the flavonoids and catechins in green tea, the green tea extract was used as the natural reducing agent to synthesize silver nanoparticles (AgNPs). Then, the obtained AgNPs were incorporated into polyvinyl alcohol/sodium alginate (PVA/SA) network to fabricate the hydrogels for antibacterial applications

In this study, the silver nanoparticles (AgNPs) were synthesized using a green one-step process and were stable in water. The extract of green Tea (Xinyang Maojian Tea) is used as a natural reducing agent to prepare the AgNPs, mainly due to that tea contains high levels of antioxidantpolyphenols, including flavonoids and catechins, which can reduce metal salts, such as gold, to the corresponding metal nanoparticles [55]. The amount of the reducing agent (as the phenolic content) in green tea extract was tested by the Folin-Ciocalteu method, with using gallic acid as a standard phenolic compound, and the result was expressed as mg/L gallic acid equivalent (GAE) [56]. Here, the total phenolic content of the prepared tea extract (Xinyang Maojian Tea) determined by this method was 3021 mg/L GAE. By changing the amount of tea extract (10 mL, 5 mL, 3 mL, 1 mL) as the reducing agent for the silver nitrate, AgNPs with different sizes and zeta potentials were obtained. Then silver-based nanocomposite hydrogels of PVA/SA/AgNPs were successfully synthesized by incorporating the synthesized AgNPs in the PVA/SA polymer matrix, as shown in **Figure 1**. The process started with the extraction of reductive metabolites from conveniently available tea leaves with hot water. The reduction of AgNO_3_ by the reductive compounds gave rise to the formation of AgNPs. Solutions of biocompatible PVA and sodium alginate were added in sequence to the monodisperse suspension of AgNPs. After a thorough mixing in darkness, the CaCl_2_ solution was added in a dropwise way to induce the formation of hydrogels [57,58].

### 3.1 TEM of the AgNPs

**Figure 2.**
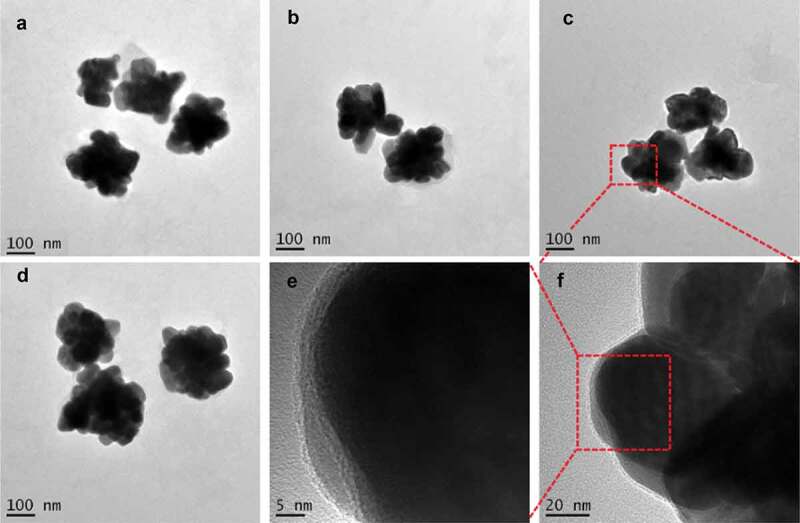
TEM images of AgNPs prepared by the reduction of AgNO_3_ with reductive compounds from tea leaves. The AgNPs had a distinct uniform interparticle separation. Images of four different preparations T-AgNPs-1 to 4 (A-D). A local area of T-AgNPs-3 was observed at two different magnifications (E, F)

TEM is an instrument generally used to characterize the size and surface topography of the synthesized nanoparticles, including AgNPs. TEM results showed that AgNPs with different sizes were synthesized by the reduction of AgNO_3_ with the tea leaf extract (**Figure 2**). At low magnifications, highly polydisperse large-size AgNPs were observed. It was apparent from the TEM image that the AgNPs had a distinct uniform interparticle separation from each other. The image of the AgNPs was close to a ‘flower shape’ with a size of about 200 nm. The shape of metal nanoparticles prepared by chemical reduction can be affected by the molecular structure of the reductive agent. The rich abundance of reductive agents in the extract of green tea led to the formation of the flower-shaped nanoparticles. Such an observed shape of the prepared nanoparticles was supportive proof that could help explain its antimicrobial activity.

### 3.2 DLS and zeta potential of the AgNPs

**Figure 3.**
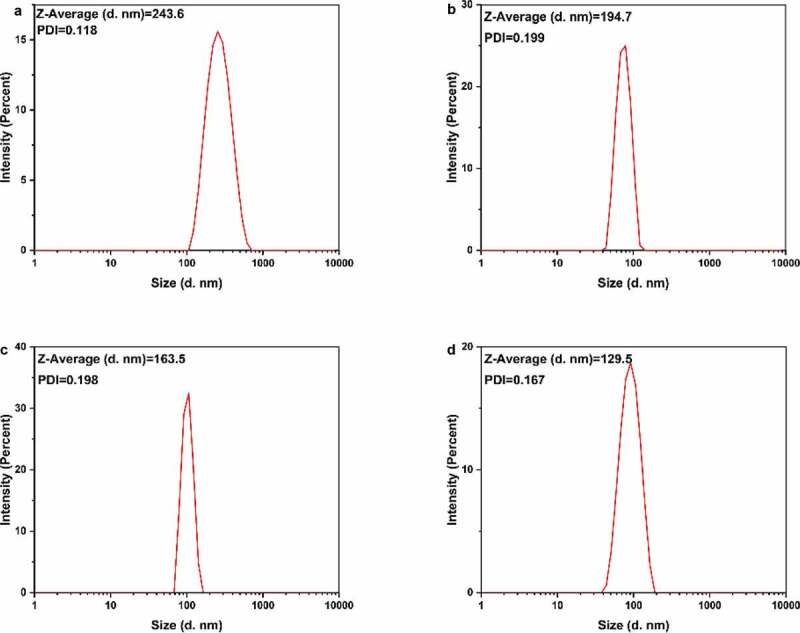
DLS analysis of the AgNPs dispersed in distilled water. The AgNPs had different average sizes, however, they could form a monodisperse suspension. T-AgNPs-1 to 4 was corresponding to A to D

DLS measures the subtle fluctuations of light intensity that occur at a resolution of a millisecond. The fluctuations are directly caused by the diffusion of molecules, which are related to the hydrodynamic radius of the molecules [59]. In the solution, AgNPs are hit by solvent molecules and thus move slowly due to Brownian motion. From this property, the diffusion rate can be determined, and the hydrodynamic radius and particle size distribution of biomolecules and nanoparticles in the solution or suspension can be measured quickly and accurately. We hypothesized that when silver nanoparticles were coated with tea phytochemicals, mainly including tea polyphenols, flavonoids, catechins, they cause significant changes in the hydrodynamic radius of T-AgNPs. The nanoparticles had a narrow particle size distribution (**Figure 3**). The hydrated particle sizes of the four nanoparticles were 243.6 nm (T-AgNPs-1), 194.7 nm(T-AgNPs-2), 163.5 nm (T-AgNPs-3), and 129.5 nm (T-AgNPs-4), respectively, indicating that tea phytochemicals were capped on the AgNPs. With the decreasing volume of tea extract during the AgNPs preparation, the hydrodynamic radius of synthesized nanoparticles decreased, proving that the tea phytochemical coated on the surface of AgNPs decreased as well. A similar phenomenon was also observed by Kim *et al* in the green synthesis of gold nanoparticles with caffeic acid. The authors attributed such an influence on the adsorption and stabilizing effect of the oxidized reducing agent [60]. Wallace R. Rolim et al. have proved that 23.7% of green tea compounds on the surface the AgNPs, which AgNPs were biogenically synthesized by using a commercial green extract, also confirming that the tea polyphenols acted as reducing and stabilizing agents for the nanoparticles. Furthermore, the polydispersity index (PDI) for the DLS is the square of the standard deviation divided by the square of the mean, and the values of PDI for different AgNPs varied, which was considered as a supplementary data to evaluate the distribution of nanoparticles. Redox processes in the reaction are dependent upon the standard reduction potentials (*E^0^*) of the reagents. The reduction potential values of plant polyphenols (*E^0^*[Ar–OH(I)/Ar–O^−^] (Ar, phenyl group), ranging from 0.3 to 0.8 V [61], are comparable to the requirement of reducing silver ions to corresponding nanoparticles (*E^0^*[Ag^+^/Ag] = 0.799 V) [62]. With increasing the volume of green tea extract, the average size of AgNPs was decreased, mainly due to that the amount of molecules on the surface of AgNPs increased, which resulted in enhancing the mono-dispersity of nanoparticles. However, stability needs to be further improved to avoid aggregation [63]. Here, Xinyang Maojian green tea was used, and the different volume of green tea extract was used to adjust the physicochemical properties of AgNPs, the flower-shaped nanoparticles were obtained, and the stability of AgNPs in water was further tested by using zeta potential test.

**Figure 4.**
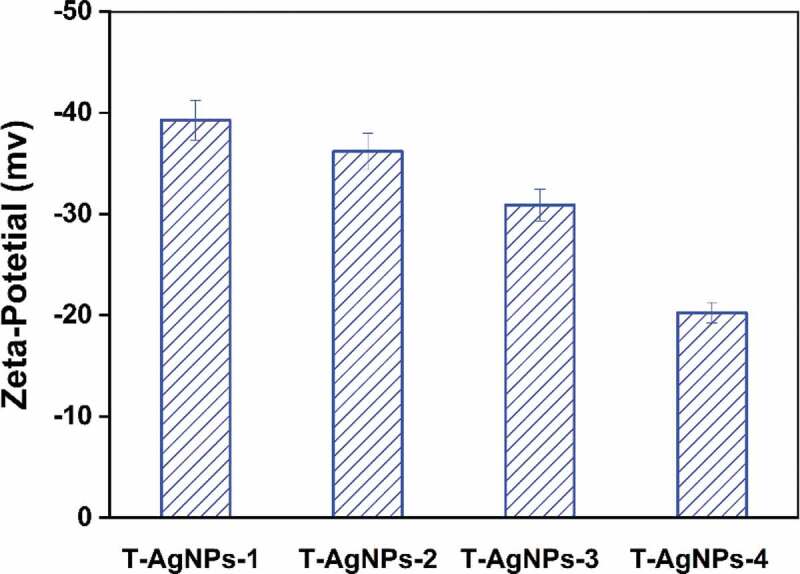
Zeta potential analysis of prepared silver nanoparticles dispersed in Millipore water. It indicated that T-AgNPs-1 and 2 had moderate stability, while T-AgNPs-3 and 4 could only form delicate dispersion

Zeta potential is an essential indicator of colloidal dispersion stability and a measure of mutual repulsion or attraction strength between particles [64]. If all particles have mutual repulsive forces, the dispersion will remain stable, while little or no repulsion will lead to aggregation. The smaller the dispersed particles, the higher the absolute value of the zeta potential (positive or negative), and the more stable the system. According to the Gouy-Chapman theory, the position of zeta potential in the diffuse double layer is close to the Gouy plane, and the negative symbol means that the net charge of the scattering object is negative [65]. We supposed that tea phytochemicals were coated on the surface of AgNPs, and the hydroxyl groups on benzene rings caused a negative charge above the isoelectric point of AgNPs. In this study, the zeta potentials were −39.3, −36.2, −30.9, −20.26, respectively, corresponding to T-AgNPs 1–4, as shown in **Figure 4**. It could be concluded that T-AgNPs-1 and T-AgNPs-2 particles exhibited less tendency to aggregate and were generally more stable due to the repelling forces amongst each other. By contrast, T-AgNPs-3 was generally stable, while T-AgNPs-4 was the most unstable and could form aggregates quickly. Usually, the zeta potentials depend on the possible reaction between the nanoparticles and the solvent, and zeta potentials are varied while changing the pH of solvent. Here, the pH of AgNPs solution was defined at around 7; thus the sample T-AgNPs-4 with the most volume of tea phytochemicals coated showed the most unstable ability under such circumstances. The obtained zeta potential data concluded that the stability of AgNPs in water could be adjusted by the volume of green tea extract during the preparation process.

### 3.3 FT-IR of silver-based nanocomposite hydrogel

**Figure 5.**
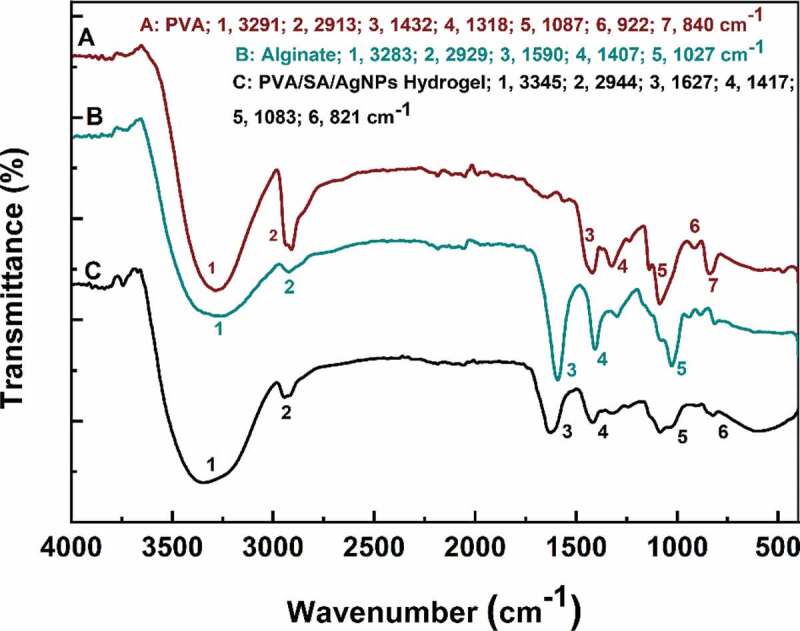
FT-IR spectra of polyvinyl alcohol, sodium alginate, and PVA/SA/AgNPs nanocomposite hydrogel (Hydrogel-1)

The FT-IR spectra of silver-based nanoparticles synthesized via green tea extract were displayed in **Figure S1** (Supporting Information). It exhibited prominent peaks at 3203, 1631, and 1383 cm^−1^ for AgNPs. Also, the peaks at 1621, 1585, 1272, 1132, and 1051 cm^−1^ were mainly corresponded to the ring breath from most of the tea catechins [66]. The FT-IR spectra of PVA, SA, and PVA/SA/AgNPs nanocomposite hydrogels were shown in **Figure 5**. The characteristic IR absorption band of PVA powder at 3291 cm^−1^ corresponded to the extension of the hydroxyl group (-OH), and the band at 2913 cm^−1^ corresponded to symmetric CH_2_ stretching, and the band at 1432 cm^−1^ corresponded to -CH_2_, respectively. The band at 1318 cm^−1^ belonged to the hydroxyl (-OH) bend and the CH swing. The band at 1087 cm^−1^ was corresponding to the C = O stretch and the hydroxyl bend. The band at 922 cm^−1^ was corresponding to the CH_2_ bend, and 840 cm^−1^ to CH swing. The infrared absorption frequency of the SA powder showed a characteristic band of 3283 cm^−1^ (hydroxyl extension), 2929 cm^−1^ (CH stretching vibration of methylene group), 1590 cm^−1^ (conjugated C = O stretching vibrations) and 1085 cm^−1^ (C-O-C) stretching vibration of sugar structure. At 1407 cm^−1^, the band was attributed to the symmetric stretching vibration of the carboxylate (C = O) ion, and the bands at 1300 cm^−1^ and 1027 cm^−1^ were C-C-H, CO stretching and C-O-C stretching of the pyranose ring, respectively [67,68]. The polyglycolic acid residue in the alginate molecule formed a chelating structure in which Ca^2+^ and polymannuronic acid residues interacted.

**Figure 6.**
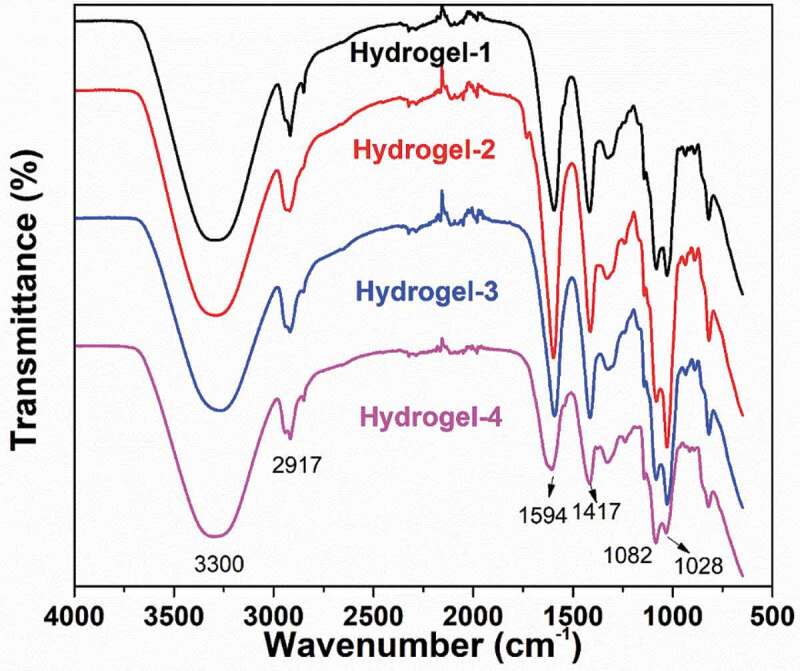
FT-IR spectra of all kinds of PVA/SA/AgNPs nanocomposite hydrogel

On the other hand, FT-IR spectra of Hydrogels-1, Hydrogels-2, Hydrogels-3, and Hydrogels-4 were shown in **Figure 6**. The IR spectra of the polymer nanocomposite with AgNPs did not show any significant binding interaction between silver and alginate, indicating that AgNPs interacted primarily with the alginate matrix by van der Waals forces.The change in intensity and shift of the IR absorption band in the PVA/SA/AgNPs nanocomposite hydrogel in the presence of silver nanoparticles indicated an interaction between PVA and SA in the silver nanoparticles. In **Figure 6**, for the PVA/SA/AgNPs nanocomposite hydrogel, the peak at 3345 cm^−1^ was corresponding to the tensile vibration of the hydroxyl groups, and the peak shift from 1627 cm^−1^ to 1598 cm^−1^ was due to C = C participation. These details indicated that the composite hydrogel was formed on the surface of AgNPs [69–71].

### 3.4 SEM of AgNPs nanocomposite hydrogel

**Figure 7.**
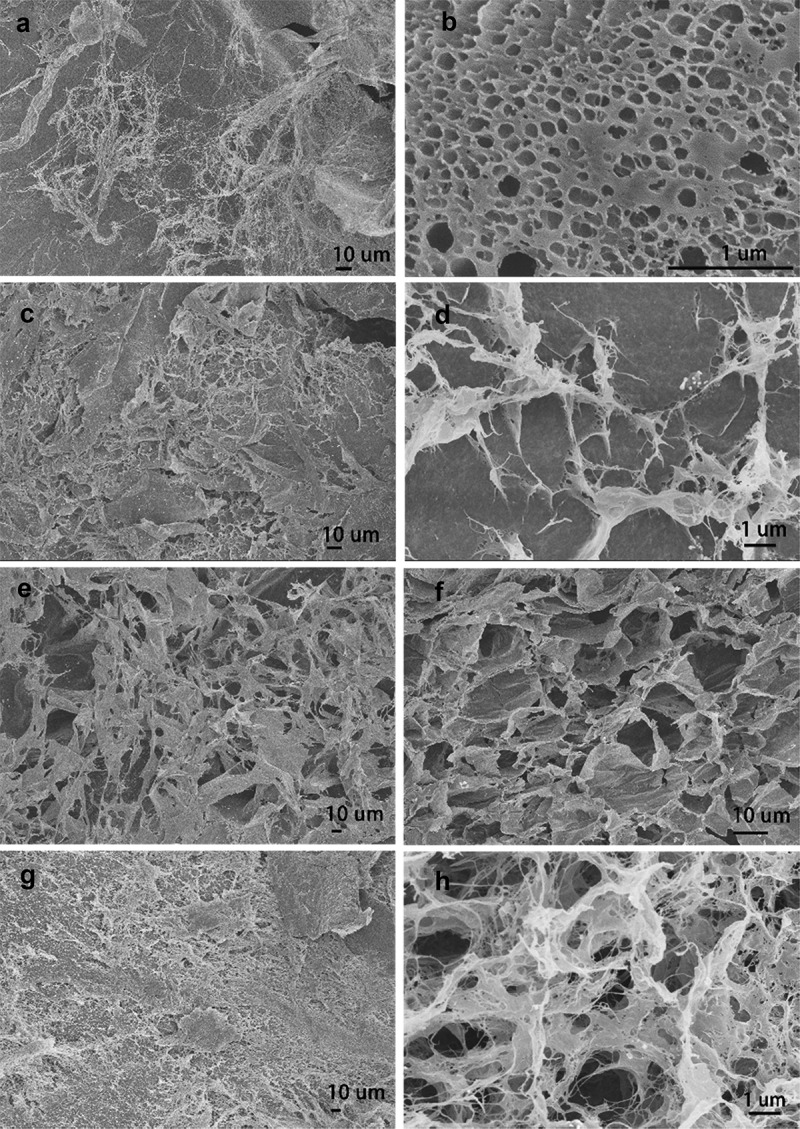
SEM images of prepared freeze-dried PVA/SA/AgNPs hydrogel. Hydrogels-1 was made from T-AgNPs-1 (A, B), and Hydrogels-2 from T-AgNPs-2 (C, D), Hydrogels-3 from T-AgNPs-3 (E, F), Hydrogels-4 from T-AgNPs-4 (G, H)

**Figure 8.**
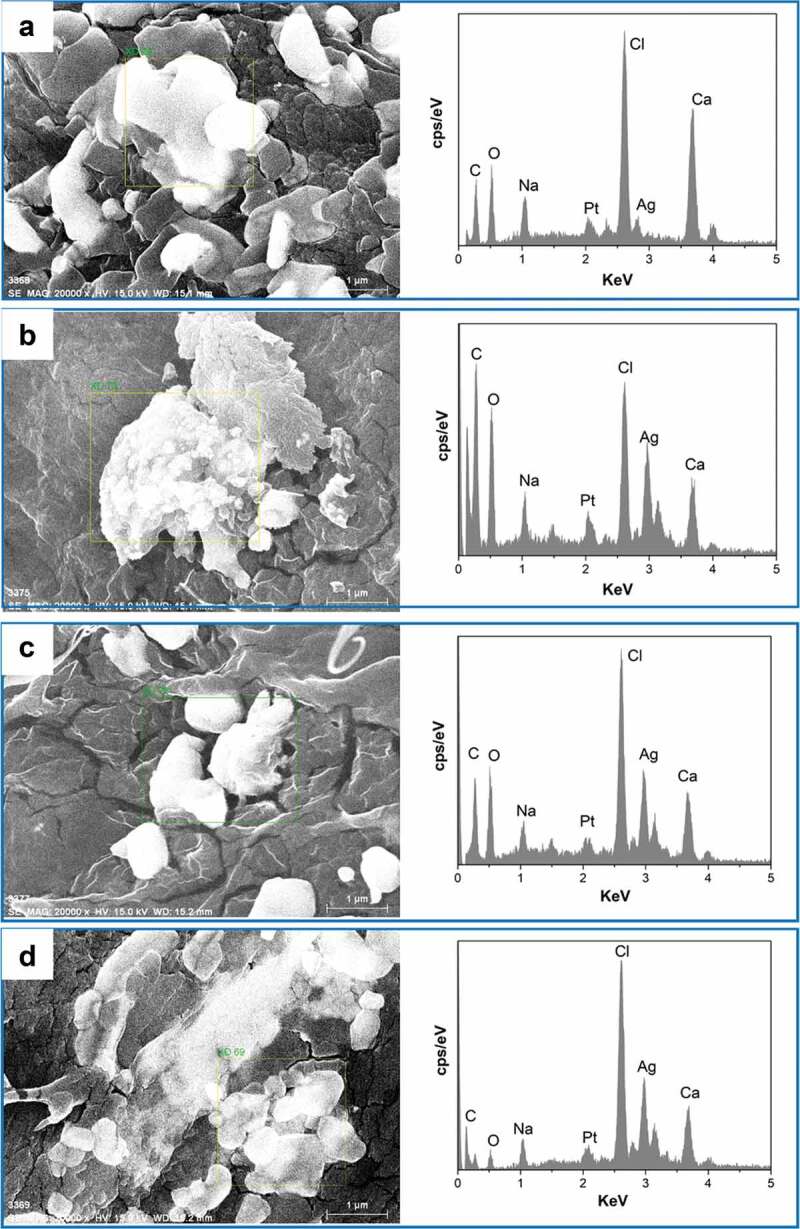
EDX analysis of prepared freeze-dried PVA/SA/AgNPs hydrogel. A: Hydrogel-1; B: Hydrogel-2; C: Hydrogel-3; D: Hydrogel-4

Scanning electron microscopy (SEM) was used to investigate the shape, size, the surface morphology and porosity of AgNPs nanocomposite hydrogels matrix, the SEM images with different magnifications for all prepared hydrogels were shown in **Figure 7**. In the PVA/SA/AgNPs nanocomposite hydrogels, micropores, and crosslinked networks were distributed on the surface uniformly. There were more uniform pores in Hydrogels-1, and for Hydrogels-2, Hydrogels-3, and Hydrogels-4, the typical porous structures were observed. Numerous wrinkles and several cavities for all samples were observed because the polymer network was collapsed incompletely during the freeze-dried. It was known that the connectivity of the pores played important role for swelling of the hydrogels fastly, and interconnected pore structure could facilitate the diffusion of the water spread through the hydrogel matrix. The size of the mesopores was rather nonhomogeneous, and there were no noticeable common features, which might be due to the formation of an amorphous structure, and the different surface porosity because of the different miscibility between PVA and SA. Furthermore, the element information and distribution of AgNPs in PVA/SA/AgNPs nanocomposite hydrogels were analyzed using Energy Dispersive X-Ray (EDX), as shown in **Figure 8**. The result confirmed that silver element was presented in all samples. The weight percent of the silver element was increased from Hydrogel-1 to Hydrogel-4, possibly due to that more tea phytochemicals coated on AgNPs, which resulted in more AgNPs were incorporated into polymer networks. The typical elements on the PVA/SA/AgNPs nanocomposite hydrogels surface are C, O, Cl, Na, and Ca were detected.

**Figure 9.**
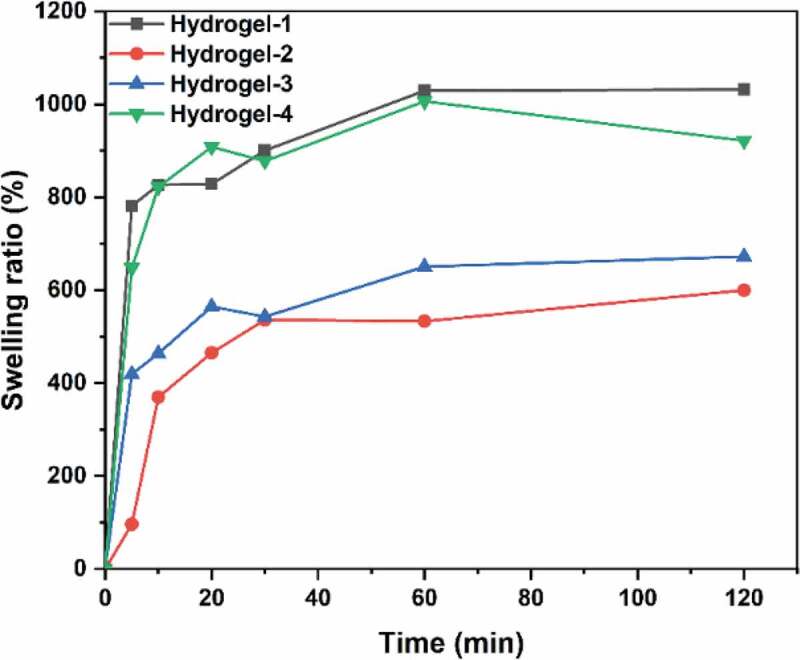
Swelling ratios of PVA/SA/AgNPs nanocomposite hydrogels. The hydrogels exhibited excellent swelling behavior. The hydrogel-1 and Hydrogels-4 had an equilibrium swelling ratio of about 900%, while hydrogel-2 and hydrogel-3 had a ratio of 500%. However, all these hydrogels could reach equilibrium in 20 min

### 3.5 Swelling behavior of the hydrogels

An ideal antimicrobial polymer/metal nanocomposite hydrogel should interact with the pathogens dispersed in water. Therefore, the swelling behavior of a hydrogel is an essential property for its application. Water absorption of PVA/SA/AgNPs nanocomposite hydrogels in aqueous solution increased rapidly in the first 20 min, and then gradually reached an equilibrium state, indicating that the porous structure had great hydrophilicity (**Figure 9**). Samples Hydrogels-1 and Hydrogels-4 showed more excellent swelling behavior than the other two AgNPs nanocomposite hydrogels, which possibly be the results of electrostatic repulsion between ionic charges in the polymer network. The presence of AgNPs might reduce the swelling ratio because of the reduced hydrophilicity, and the increase in AgNPs content also reduced the crosslink density. The different percentage of AgNPs in the silver-based nanocomposite gel changed the surface charge and thus affected its swelling ability, as well as the antibacterial properties. The antimicrobial activities of these biomaterials primarily depend on the silver ions released from the hydrogels. The swelling properties will affect the release rate and process and thus the apparent antimicrobial activity: a low swelling ratio will support a long period of release, and vise versa. However, hydrogel with a higher swelling will release more silver ions in a short period, exhibiting a stronger antimicrobial activity because antimicrobial activity is generally measured within 24 hours.

**Figure 10.**
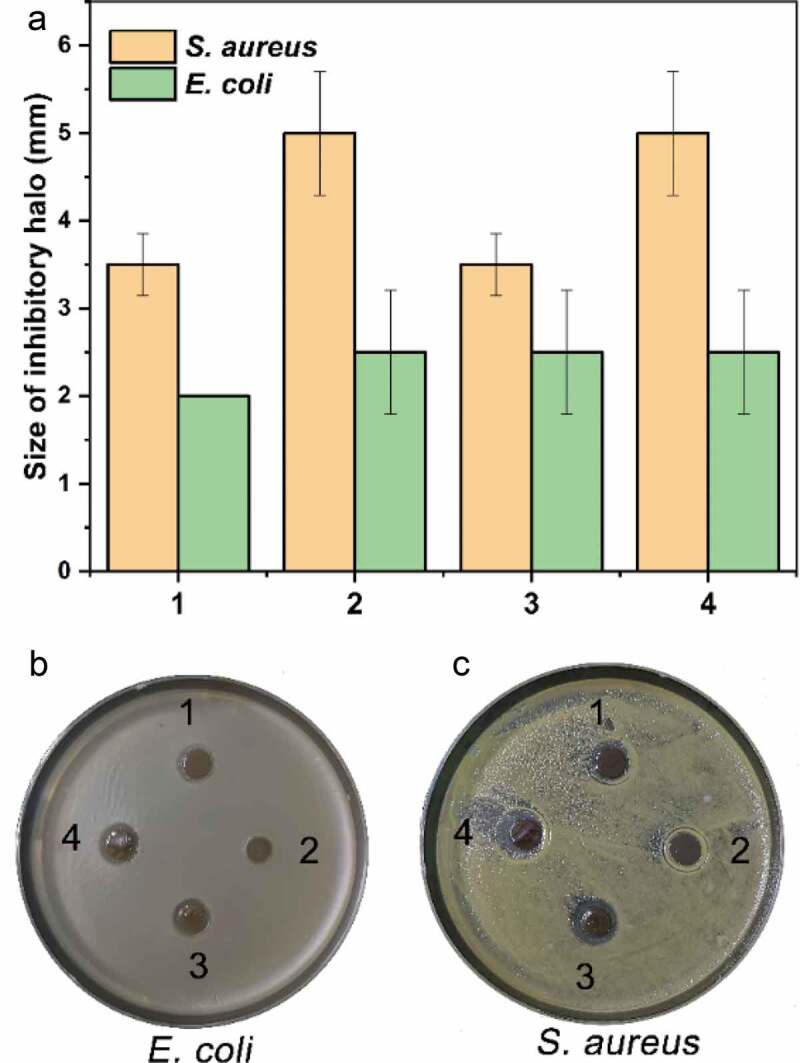
The antimicrobial activity of the hydrogels. The hydrogels had a different growth inhibitory effect on the growth of Gram-positive (*E. coli*) and Gram-negative (*S. aureus*) bacteria, which was indicated by the size of the inhibitory halo (A) and photos (B and C). By comparison, the hydrogels had a relatively more substantial inhibitory effect on the Gram-positive bacterium than on the negative

### 3.6 Antimicrobial activity of the PVA/SA/AgNPs nanocomposite hydrogels

All four hydrogels exhibited antimicrobial activities on the growth of typical Gram-positive and Gram-negative bacteria in the antimicrobial activity assay of these hydrogels, and the results were shown in **Figure 10**. By contrast, these hydrogels exhibited a more substantial inhibitory effect on Gram-positive bacterium *S. aureus*. In the preparation of the four hydrogels, different volumes of tea extract were used. From the results of the antimicrobial assay, we could see that, although tea extract was indispensable for the preparation of AgNPs, it had a negative effect on the overall antimicrobial activity of the hydrogel, implying an optimization of the utilization of tea extract regarding the antibacterial activity should be carried out in the future studies. Furthermore, the optical density at 600 nm (OD600) of both bacterial suspension after growth for 8 h culturing with hydrogels was tested, as shown in **Figure S2** (Supporting Information), the results were consistent with the inhibitory halo results, also confirmed that the hydrogels could efficiently inhibit the proliferation of typical Gram-positive and Gram-negative bacteria in comparison with control group.

The antibacterial activity of silver has long been known and investigated [72]. The general belief that silver has considerably lower toxicity to human cells than to bacteria laid the fundamental basis of its diverse applications [73]. The effect of silver ion on the growth of a specific microbe is, in essence, the overall outcome of the complicated interactions between the ion and the cellular structure or components. Therefore, the observations of the bactericidal activity of silver ion or bacterial resistance to silver are stringently case-dependent [72]. Regarding the effect of AgNPs on bacterial growth, some additional factors such as size [74] and shape [75] could also play a role. Ivask *et al*. tested the growth-inhibiting effect of silver nanoparticles with different sizes (10, 20, 40, 60, and 80 nm). They found that the size could affect the release of Ag ions from the particle and thus the bioavailability of Ag ions to the cell of *E. coli* if the size was in the range of 20–80 nm. However, AgNPs of 10 nm exhibited more potent inhibition of growth than the AgNO_3_ by providing a higher bioavailability. Also, the shape of nanoparticles could influence the release rate of Ag ions and thus the antimicrobial activities. Fructose was reported to enhance antimicrobial activities [76]. The observed inhibitory effect of hydrogels was weaker than those of our previous study [49], which might be explained by the lowered release rate of Ag ion due to the restriction from the polymers on the surface of the particles. However, because of the same reason, the hydrogel exhibited an extended period of inhibition on bacterial growth: the halos were quite noticeable even when the lawn was formed due to the growth of bacteria around the hydrogel slices (**Figure 10**). Studies on the cellular effects of AgNPs treatment on the drug-resistant G^−^ *Pseudomonas aeruginosa* revealed that AgNPs could cause oxidative stress similar to that of antibiotics, characterized by the upregulated expression of enzymes such as superoxide dismutase, catalase, and peroxidase as a response to combat the excessive production of reactive oxidative species [77]. In a combined utilization of AgNPs and antibiotics, enhanced antimicrobial activity of antibiotics was observed due to the impact of AgNPs on the cell membrane [78]. The significant differences in the thickness and structure of the cell wall of G^+^ and G^−^ bacteria determine the accessibility of the Ag ions released from the AgNPs and hydrogels, and thus the different growth inhibitory effects.

## 4. Conclusions

In this study, AgNPs were successfully synthesized by the reduction of AgNO_3_ with reductive compounds extracted from the local agricultural produce tea leaves with hot water. To extend the application scope of AgNPs, we prepared and characterized the silver-based PVA/SA nanocomposite hydrogels. The AgNPs prepared with the tea extract were immobilized in the polymer network by ionic and physical cross-linking and effectively limit the free diffusion of AgNPs from the matrix. High hydrophilicity conferred the silver-based nanocomposite hydrogel excellent swelling property. The content of AgNPs present in gel could change the surface charge and affect its swelling behavior. These gels exhibited good antimicrobial activity on typical G^+^ and G^−^ bacteria, implying the potential applications of these silver nanocomposite hydrogels as antibacterial agents in related areas.
